# Supplementary material for: Modeling glioblastoma heterogeneity as a dynamic network of cell states
Source: Mol Syst Biol. 2021 Sep 16;17(9):e10105. doi: 10.15252/msb.202010105 (PMC8444284; doi:10.15252/msb.202010105)
Supplement: Supplementary file 6 — Source Data for Figure 5 [file MSB-17-e10105-s004.zip › Figure5A_sourcedata/GSEA_3071/hallmarks_stateII.GseaPreranked.1623742536692/HALLMARK_GLYCOLYSIS.html]

Details for gene set HALLMARK\_GLYCOLYSIS[GSEA]

|  || Dataset | state23071 |
| Phenotype | NoPhenotypeAvailable |
| Upregulated in class | na\_pos |
| GeneSet | HALLMARK\_GLYCOLYSIS |
| Enrichment Score (ES) | 0.20748843 |
| Normalized Enrichment Score (NES) | 1.149409 |
| Nominal p-value | 0.2470024 |
| FDR q-value | 0.33015704 |
| FWER p-Value | 0.997 |
Table: GSEA Results Summary

  

Fig 1: Enrichment plot: HALLMARK\_GLYCOLYSIS      
 Profile of the Running ES Score & Positions of GeneSet Members on the Rank Ordered List

  

| PROBE | GENE SYMBOL | GENE\_TITLE | RANK IN GENE LIST | RANK METRIC SCORE | RUNNING ES | CORE ENRICHMENT || 1 | CDK1 |  |  | 14 | 1.540 | 0.0362 | Yes |
| 2 | DEPDC1 |  |  | 126 | 0.999 | 0.0344 | Yes |
| 3 | PFKP |  |  | 136 | 0.988 | 0.0576 | Yes |
| 4 | MET |  |  | 183 | 0.897 | 0.0692 | Yes |
| 5 | EGFR |  |  | 198 | 0.874 | 0.0883 | Yes |
| 6 | QSOX1 |  |  | 204 | 0.870 | 0.1095 | Yes |
| 7 | TFF3 |  |  | 225 | 0.851 | 0.1264 | Yes |
| 8 | CENPA |  |  | 303 | 0.751 | 0.1266 | Yes |
| 9 | AURKA |  |  | 360 | 0.705 | 0.1309 | Yes |
| 10 | CLN6 |  |  | 431 | 0.663 | 0.1306 | Yes |
| 11 | HMMR |  |  | 475 | 0.639 | 0.1364 | Yes |
| 12 | NT5E |  |  | 481 | 0.637 | 0.1516 | Yes |
| 13 | ENO1 |  |  | 501 | 0.629 | 0.1631 | Yes |
| 14 | COL5A1 |  |  | 556 | 0.599 | 0.1651 | Yes |
| 15 | PYGL |  |  | 636 | 0.563 | 0.1600 | Yes |
| 16 | CYB5A |  |  | 737 | 0.520 | 0.1485 | Yes |
| 17 | NASP |  |  | 741 | 0.518 | 0.1611 | Yes |
| 18 | ERO1A |  |  | 767 | 0.510 | 0.1681 | Yes |
| 19 | FKBP4 |  |  | 806 | 0.495 | 0.1714 | Yes |
| 20 | SLC16A3 |  |  | 811 | 0.494 | 0.1831 | Yes |
| 21 | ME1 |  |  | 835 | 0.489 | 0.1900 | Yes |
| 22 | ADORA2B |  |  | 841 | 0.486 | 0.2013 | Yes |
| 23 | SDC1 |  |  | 872 | 0.479 | 0.2061 | Yes |
| 24 | PYGB |  |  | 916 | 0.467 | 0.2075 | Yes |
| 25 | IER3 |  |  | 1010 | 0.442 | 0.1958 | No |
| 26 | TXN |  |  | 1087 | 0.419 | 0.1877 | No |
| 27 | TPI1 |  |  | 1111 | 0.415 | 0.1927 | No |
| 28 | POLR3K |  |  | 1140 | 0.410 | 0.1963 | No |
| 29 | STMN1 |  |  | 1249 | 0.387 | 0.1794 | No |
| 30 | PLOD1 |  |  | 1292 | 0.380 | 0.1788 | No |
| 31 | G6PD |  |  | 1346 | 0.368 | 0.1751 | No |
| 32 | LDHA |  |  | 1374 | 0.361 | 0.1777 | No |
| 33 | P4HA2 |  |  | 1398 | 0.358 | 0.1812 | No |
| 34 | GALE |  |  | 1476 | 0.344 | 0.1709 | No |
| 35 | PRPS1 |  |  | 1620 | 0.320 | 0.1437 | No |
| 36 | PGAM1 |  |  | 1659 | 0.316 | 0.1424 | No |
| 37 | PAXIP1 |  |  | 1673 | 0.313 | 0.1472 | No |
| 38 | NANP |  |  | 1760 | 0.304 | 0.1337 | No |
| 39 | PGM2 |  |  | 1783 | 0.301 | 0.1359 | No |
| 40 | VCAN |  |  | 1793 | 0.299 | 0.1414 | No |
| 41 | SDC3 |  |  | 1908 | 0.284 | 0.1204 | No |
| 42 | TPBG |  |  | 1914 | 0.284 | 0.1265 | No |
| 43 | SRD5A3 |  |  | 1996 | 0.273 | 0.1134 | No |
| 44 | PPIA |  |  | 2072 | 0.265 | 0.1016 | No |
| 45 | PGK1 |  |  | 2126 | 0.260 | 0.0951 | No |
| 46 | UGP2 |  |  | 2141 | 0.258 | 0.0983 | No |
| 47 | PKM |  |  | 2199 | 0.252 | 0.0906 | No |
| 48 | PPP2CB |  |  | 2394 | -0.270 | 0.0494 | No |
| 49 | CHST12 |  |  | 2421 | -0.273 | 0.0500 | No |
| 50 | NOL3 |  |  | 2443 | -0.276 | 0.0519 | No |
| 51 | GNPDA1 |  |  | 2511 | -0.287 | 0.0426 | No |
| 52 | SDC2 |  |  | 2574 | -0.297 | 0.0349 | No |
| 53 | GPC4 |  |  | 2577 | -0.298 | 0.0421 | No |
| 54 | HS2ST1 |  |  | 2620 | -0.304 | 0.0394 | No |
| 55 | COPB2 |  |  | 2661 | -0.310 | 0.0375 | No |
| 56 | GLRX |  |  | 2834 | -0.342 | 0.0036 | No |
| 57 | ZNF292 |  |  | 2882 | -0.352 | 0.0010 | No |
| 58 | MXI1 |  |  | 3000 | -0.372 | -0.0185 | No |
| 59 | CAPN5 |  |  | 3018 | -0.378 | -0.0130 | No |
| 60 | AGL |  |  | 3134 | -0.410 | -0.0310 | No |
| 61 | SOX9 |  |  | 3230 | -0.435 | -0.0434 | No |
| 62 | B3GAT3 |  |  | 3258 | -0.443 | -0.0387 | No |
| 63 | HSPA5 |  |  | 3335 | -0.468 | -0.0455 | No |
| 64 | PGLS |  |  | 3432 | -0.513 | -0.0561 | No |
| 65 | VEGFA |  |  | 3532 | -0.560 | -0.0663 | No |
| 66 | B3GAT1 |  |  | 3677 | -0.668 | -0.0848 | No |
| 67 | PAM |  |  | 3685 | -0.671 | -0.0693 | No |
| 68 | CXCR4 |  |  | 3733 | -0.710 | -0.0627 | No |
| 69 | CHPF |  |  | 3820 | -0.808 | -0.0632 | No |
| 70 | IDH1 |  |  | 3842 | -0.832 | -0.0470 | No |
| 71 | CTH |  |  | 3970 | -1.112 | -0.0499 | No |
| 72 | STC1 |  |  | 4026 | -1.424 | -0.0268 | No |
| 73 | IGFBP3 |  |  | 4058 | -1.726 | 0.0099 | No |
Table: GSEA details [plain text format]

  

Fig 2: HALLMARK\_GLYCOLYSIS: Random ES distribution      
 Gene set null distribution of ES for **HALLMARK\_GLYCOLYSIS**
